# The TMS Map Scales with Increased Stimulation Intensity and Muscle Activation

**DOI:** 10.1007/s10548-015-0447-1

**Published:** 2015-09-04

**Authors:** Mark van de Ruit, Michael J. Grey

**Affiliations:** NIHR Surgical Reconstruction and Microbiology Research Centre, School of Sport, Exercise and Rehabilitation Sciences, University of Birmingham, Edgbaston, B15 2TT UK; MRC-ARUK Centre for Musculoskeletal Ageing Research, School of Sport, Exercise and Rehabilitation Sciences, College of Life and Environmental Sciences, University of Birmingham, Edgbaston, B15 2TT UK

**Keywords:** Mapping, Stimulation intensity, Muscle activation, TMS, Corticospinal excitability

## Abstract

One way to study cortical organisation, or its reorganisation, is to use transcranial magnetic stimulation (TMS) to construct a map of corticospinal excitability. TMS maps are reported to be acquired with a wide variety of stimulation intensities and levels of muscle activation. Whilst MEPs are known to increase both with stimulation intensity and muscle activation, it remains to be established what the effect of these factors is on the map’s centre of gravity (COG), area, volume and shape. Therefore, the objective of this study was to systematically examine the effect of stimulation intensity and muscle activation on these four key map outcome measures. In a first experiment, maps were acquired with a stimulation intensity of 110, 120 and 130 % of resting threshold. In a second experiment, maps were acquired at rest and at 5, 10, 20 and 40 % of maximum voluntary contraction. Map area and map volume increased with both stimulation intensity (*P* < 0.01) and muscle activation (*P* < 0.01). Neither the COG nor the map shape changed with either stimulation intensity or muscle activation (*P* > 0.09 in all cases). This result indicates the map simply scales with stimulation intensity and muscle activation.

## Introduction

Transcranial magnetic stimulation (TMS) maps of the primary motor cortex have been used to non-invasively study brain organisation and brain topography. The TMS map is created by stimulating at different sites across the motor cortex, combining the position of every stimulus with the size of the recorded motor evoked potentials (MEPs) (Wassermann et al. 1992; Wilson et al. 1993). Recently, we presented a method to acquire data for the TMS maps that reduces acquisition time to 2 min (van de Ruit et al. 2015). Whilst the MEP increases with a higher stimulation intensity and greater muscle activation (Day et al. 1989; Hess et al. 1987; Kiers et al. 1993; Rothwell et al. 1991), it is unknown what happens with the TMS map’s centre of gravity (COG), map area and map volume.

In one of the early studies using TMS mapping, Wasserman et al. (1992) used a stimulation intensity of 100 % of the maximum stimulator output (MSO). Although 100 % MSO may be required in clinical studies where MEPs are small, a stimulation intensity of 110–120 % of resting motor threshold (RMT) is more commonly used in healthy participants (e.g. Classen et al. 1998; Pascual-Leone et al. 1995; Uy et al. 2002). Higher stimulation intensities are associated with stronger magnetic fields; thereby stimulating a greater area of the cortex including deeper lying structures (Day et al. 1989). Whereas MEP amplitude increases with higher stimulation intensities, the amplitude saturates when the intensity is high enough. This can be clearly observed when constructing recruitment curves, plotting the stimulation intensity versus MEP amplitude (Devanne et al. 1997). Nonetheless, higher stimulation intensities are associated with a greater area of the cortex resulting in MEPs (Thordstein et al. 2013), but it remains unclear how stimulation intensity affects the COG and map volume.

Not only stimulation intensity but also muscle activation at the time of administering TMS is correlated with MEP magnitude (Hess et al. 1987; Kiers et al. 1993). In contrast to the effect of stimulation intensity, for which its effect on the TMS map has not been systematically examined, it has been documented that muscle activation leads to a greater map area and translation of the COG compared with a map acquired when the muscle is relaxed (Wilson et al. 1995). However, not all groups report that COG moves when the muscle is activated (Classen et al. 1998; Ngomo et al. 2012). Moreover, when acquiring the map at a stimulation intensity relative to active motor threshold instead of resting motor threshold, the map area does not change with muscle activation (Ngomo et al. 2012). Mostly, TMS maps are created either when the muscle is at rest (e.g. Pascual-Leone et al. 1995; Wassermann et al. 1992) or slightly active, usually between 5 and 10 % of the maximum voluntary contraction (MVC) (Byrnes et al. 1999; Wilson et al. 1993). At voluntary muscle activation greater than 10 % of MVC, MEP amplitude for a small hand muscle has been reported to saturate (Helmers et al. 1989; Taylor et al. 1997). However, no study has investigated the effect of different levels of muscle activity on the TMS map when muscle activation exceeds 10 % of MVC.

Frequently, the map is elongated along the main coil axis (Wilson et al. 1993), but it is unclear if the map’s shape remains unchanged when stimulating at higher intensities or when the cortex is more excitable during muscle activation. Quantifying the map’s shape might be of interest when brain reorganisation is studied, and has never been explored. Therefore, in this study the map shape was used as a novel measure to quantify the TMS map.

The aim of this study was to describe the effects of stimulation intensity and different levels of muscle activation on map outcome parameters: COG, map area, map volume and map shape. As the stimulated cortical area scales with stimulation intensity (Thielscher and Kammer 2004), we hypothesized an increase in map area and volume whilst COG and map shape remains unaffected. As the MEP response saturates when the muscle is activated above 10 % MVC, we hypothesized that map area and map volume would also saturate when the muscle activity exceeds this level, with no change in COG and map shape.

## Methods

### Participants

In total, 16 healthy participants were recruited for the study with some participating in both experiments (Experiment 1; 12 participants: 23 ± 3 years, range 20–29, 6 female; Experiment 2; 12 participants: 23 ± 3 years, range 20–28, 3 female). Participants were screened for contraindications to TMS using a modified version of the TMS adult safety questionnaire originally suggested by Keel et al. (2001). All participants provided written informed consent. The study was approved by the University of Birmingham’s Science, Technology, Engineering and Mathematics ethics committee (ERN_12-1189), and all experiments were performed in accordance with the Declaration of Helsinki.

### Electromyography

Bipolar surface electrodes (Blue Sensor N, Ambu, Denmark) were used to record the electromyographic (EMG) activity of the first dorsal interosseus (FDI). All EMG signals were amplified (500–2 k), band pass filtered (20–1000 Hz), and digitally sampled at 5 kHz to be stored for offline analysis.

### Transcranial Magnetic Stimulation

Magnetic stimulation was delivered with a Magstim Rapid^2^ (Magstim Ltd, Dyfed, United Kingdom) and a custom made polyurethane coated 90 mm figure-of-8 coil (type: batwing; Type No. 15411). The coil was held tangentially to the scalp and orientated at 45° to the midline with the handle pointing posteriorly (Brasil-Neto et al. 1992). The stimulation site evoking the largest MEP, was found by repeated stimulation approximately every 2 s during which the EMG was visually inspected. Whilst holding the coil over the hotspot, resting motor threshold (RMT) was determined as the intensity at which at least 5 out of 10 stimuli evoked MEPs with a peak-to-peak amplitude of greater than 50 µV (Groppa et al. 2012; Rossini et al. 1994). Coil position and orientation were monitored throughout the experiment using frameless stereotaxy (BrainSight2, Rogue Research Inc, Montreal, Canada).

## Experimental Protocol

The participants were seated comfortably in a chair with the right hand resting pronated on a table and the distal phalanx of the index finger fixed to a force transducer. Each TMS map was created from 80 stimuli using an interstimulus interval of 1.5 s, pseudorandomly applied in a 6 × 6 cm grid, using the rapid mapping technique described by van de Ruit et al. (2015). Excitability maps were constructed and analysed offline. Map COG, area, volume, and shape were calculated (see “Data Analysis” section below).

### Experiment 1: Effect of Stimulation Intensity

To study the effect of stimulation intensity, maps were created from 12 participants at 110, 120 and 130 % of resting motor threshold (RMT). The participants were instructed to keep their hand fully relaxed during the experiment. Online feedback of FDI EMG was provided to ensure compliance with this instruction and to focus their attention as the stimuli were being delivered. Three maps were acquired at each stimulation intensity, with the order of presentation randomised.

### Experiment 2: Effect of Muscle Activation

To study the effect of muscle activation, maps were created from 12 participants with the FDI muscle activated at 5, 10, 20 or 40 % of their MVC and when relaxed. TMS maps were constructed at each level of muscle activation using a stimulation intensity of 120 % RMT. The force exerted by abduction of the FDI was measured by a cantilever beam load cell (NL 62–50 kg, Digitimer Ltd, Welwyn Garden City, UK). The participant’s MVC force was determined during three consecutive trials, with a 30 s rest period between trials. The force feedback signal was low-pass filtered at 1 Hz with a second order Butterworth filter. The participant was instructed to maintain a steady force throughout the mapping procedure. Visual feedback of the force signal was provided on a monitor in direct line of sight of the participant. A single bar was presented with a horizontal target line and two additional horizontal lines to denote a window that was 10 % of the target force. Whenever the force was outside this target window, the bar turned red to indicate the force exerted was not in the target window. Three maps were collected for each level of muscle activation, with order of presentation randomised. To prevent muscle fatigue, a rest period of at least 2 min was used between each map.

## Data Analysis

### Creating the Map

**Fig. 1:**
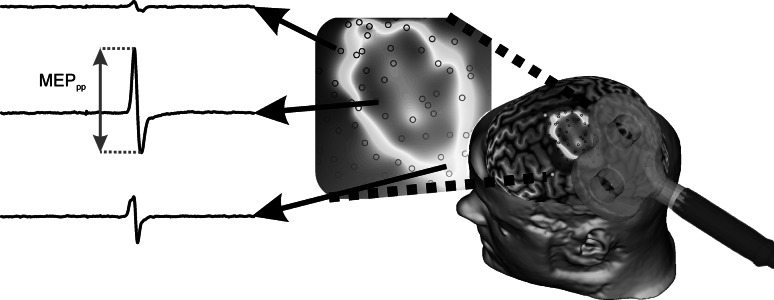
An illustration outlining the creation of a TMS map. A 6 × 6 cm square grid is defined in the neuronavigation software (BrainSight 2.0, Rogue Research) and each stimulation site is matched with the recorded EMG. The motor evoked potential’s peak-to-peak (MEP_pp_) value is extracted from each EMG recording. Using a bespoke MATLAB script, the 3D position data are then matched with the MEP_pp_ data to fit a surface and visualise the resulting TMS map in a 2D plane

Figure 1 illustrates how the EMG and neuronavigation data were used to construct a TMS map. The stimulation position was extracted from the neuronavigation data and transposed into a 2D plane. The corresponding MEP observed in the EMG was quantified by its peak-to-peak value (MEP_pp_), which was extracted from a window between 20 and 50 ms after stimulation. All MEPs were normalised to the electrically evoked maximal M-wave (M_max_). To obtain the M_max_, a bipolar probe was used to stimulate the median nerve at the level of the elbow using a constant current stimulator (Digitimer DS7A, Digitimer Ltd, Welwyn Garden City, UK).

Analysis was performed offline with a bespoke MATLAB script (MATLAB Release 2012b, The MathWorks, Inc., Natick, Massachusetts, United States) to create a full 2D surface TMS map, using an approximant fitting function (‘gridfit’ D’Errico 2005). Individual stimuli within a map were excluded from the analysis if the stimulation or corresponding MEP did not fulfil one of four conditions: (1) the root mean square value of the background EMG (50–5 ms before stimulation) was within Mean ± 2 *SD* of all stimuli; (2) stimulation at most 10 mm outside the grid border; (3) MEP size not larger than Mean ± 3.5 *SD* of all MEPs in the map; (4) angle and translation of stimulus within the 99 % predication interval of all stimuli. All maps were created with the same colour axis so that differences could be easily observed.

### Map Parameters

Maps were characterised by COG, map area, map volume and map shape. The map area was defined as the part of the map where the MEP_pp_ exceeded a predefined threshold. In Experiment 1 this threshold was set to 10 % of the maximum MEP_pp_ as recorded in the 110 % RMT condition. For Experiment 2 the threshold was chosen as 10 % of the maximum MEP_pp_ for the maps created in the 5 % of MVC condition. These thresholds were chosen based on the lowest stimulation intensity condition (110 % RMT) and muscle activation condition (5 % of MVC) to enable appropriate characterisation of the effect of increasing stimulation intensity or greater muscle activation on the map. The stimulation points and their corresponding MEP_pp_ values were used to approximate a 6 × 6 cm grid composed of 2500 pixels using MATLAB’s ‘gridfit’ function (D’Errico 2005). Next, the number of pixels with an approximated MEP_pp_ amplitude greater than the 10 % threshold was calculated, and expressed as total map area (in mm^2^). The map volume was determined by the sum of all MEP_pp_ exceeding the same threshold, normalised to the maximum volume of all maps in a session. The maps COG x- and y-coordinate was calculated by using the MEP_pp_ amplitude and its position on the map, creating an amplitude weighted mean of the map. Full details of this process are described in van de Ruit et al. (2015). Finally, in Experiment 2, we quantified the order of COG translation as a result of muscle activation by calculating the Euclidian distance between the COGs during all active conditions with the mean COG in the resting condition.

In addition to these traditional measures, we defined an extra measure to quantify the map shape: the aspect ratio. The aspect ratio is characterised by the ratio of the major and minor axes of a fitted ellipse and was defined to describe the expansion of the excitable area. The ellipse was fitted through the points that defined the positions where the MEP_pp_ amplitude fell below the 10 % threshold. By choosing the 10 % cut-off, the ellipse roughly outlines an area similar to the area parameter. The cut-off was increased to 30 % for Experiment 2 because the increased muscle activation produced much larger MEPs and, in many cases, the 10 % cut-off resulted in an inability to fit an ellipse because it would fall outside the border of the map.

## Statistical Analysis

Statistical testing was conducted with NCSS 2007 v07.1.4. Tests were considered significant at α = 0.05. As the descriptive statistics showed much of the data violated the standard assumptions of normality (typical positively skewed or uniformly distributed) and equal variance, all statistical tests were conducted with non-parametric tests.

### Experiment 1: Effect of Stimulation Intensity

All parameters (area, volume, xCOG, yCOG and aspect ratio) were compared between stimulation intensities using the non-parametric Friedman Test. Post-hoc comparisons were performed using the Wilcoxon Signed-Rank Test. A Bonferroni adjustment was applied to compensate for the multiple comparisons; therefore, in this case α = 0.017 (3 comparisons) was used for significance.

### Experiment 2: Effect of Muscle Activation

All parameters (area, volume, xCOG, yCOG and aspect ratio) were compared using the non-parametric Friedman Test across all conditions with muscle activity. Post-hoc comparisons were performed using the Wilcoxon Signed-Rank Test. A Bonferroni adjustment was applied to compensate for the multiple comparisons; therefore, in this case α = 0.0083 (6 comparisons) was used for significance.

## Results

### Data Exclusion

All participants tolerated the TMS well and completed the study. Stimuli were excluded from the analysis based on high background EMG, or incorrect coil position and/or orientation relative to the grid. In total 8.0 % of all stimuli were excluded before analysing the maps (285 maps analysed). Most stimuli were excluded based on a high background EMG (4.2 %) or angle and translation of the stimulus with respect to the skull (3.3 %). On average, a median number of 6 stimuli were excluded for each participant (inter quartile range: 5–8).

### Experiment 1: Effect of Stimulation Intensity

**Fig. 2:**
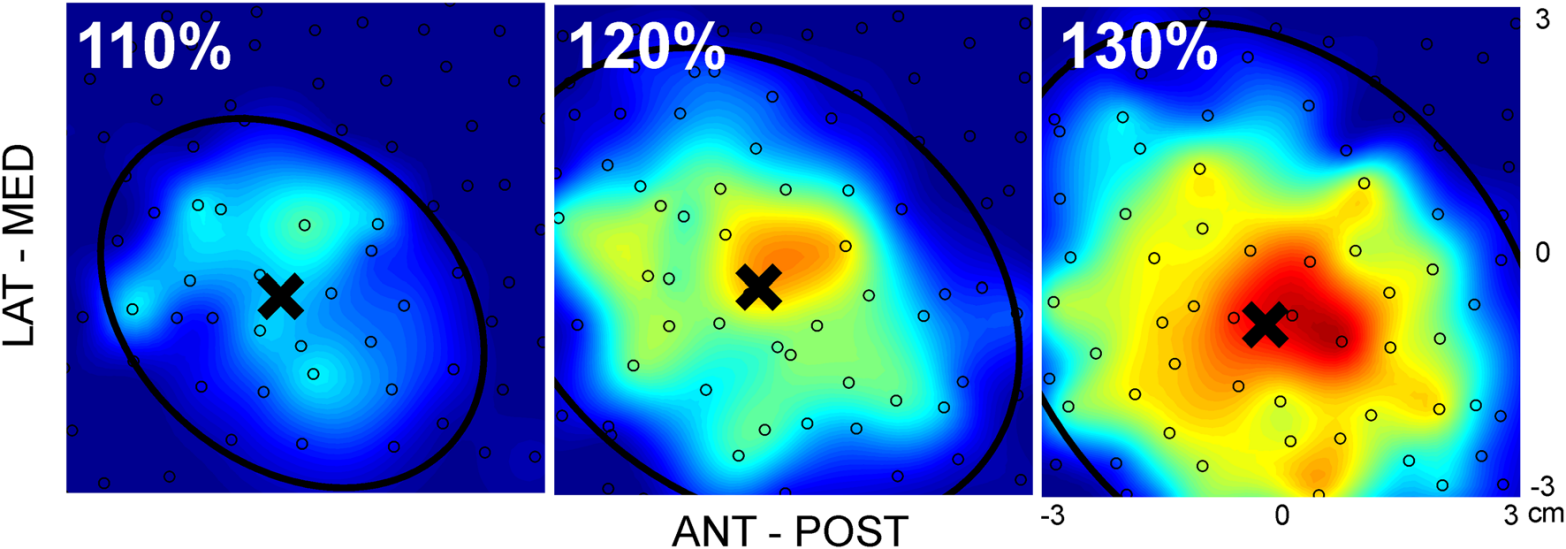
Single participant data illustrating TMS maps acquired at three different stimulation intensities (110, 120 and 130 % of resting motor threshold) using a 6 × 6 cm grid and 80 stimuli with a 1.5 s interstimulus interval. Each * black open circle* represents one stimulus. The size of the approximated MEP_pp_ is indicated by the * colour*, with * blue* representing a small MEP_pp_ and * red* representing the greatest MEP_pp_. The *black cross* (×) highlights the centre of gravity. In this participant, stimulation intensity was found not to affect the x- or y-coordinate of the centre of gravity, however map area and volume significantly increased with stimulation intensity. An ellipse was fitted through the data points representing 10 % of the maximum MEP within the 110 % maps and used to study changes in the shape of excitable area of the map. No change in the shape of the ellipse was found (Color figure online)

Three different stimulation intensities (110, 120 and 130 % of RMT) were used to examine the effect of the stimulation intensity on the excitability maps. Data from a representative participant are shown in Fig. 2.

In this case it can be clearly observed that the cortical representation scales with stimulation intensity, whilst the COG and aspect ratio are unaffected.

**Fig. 3:**
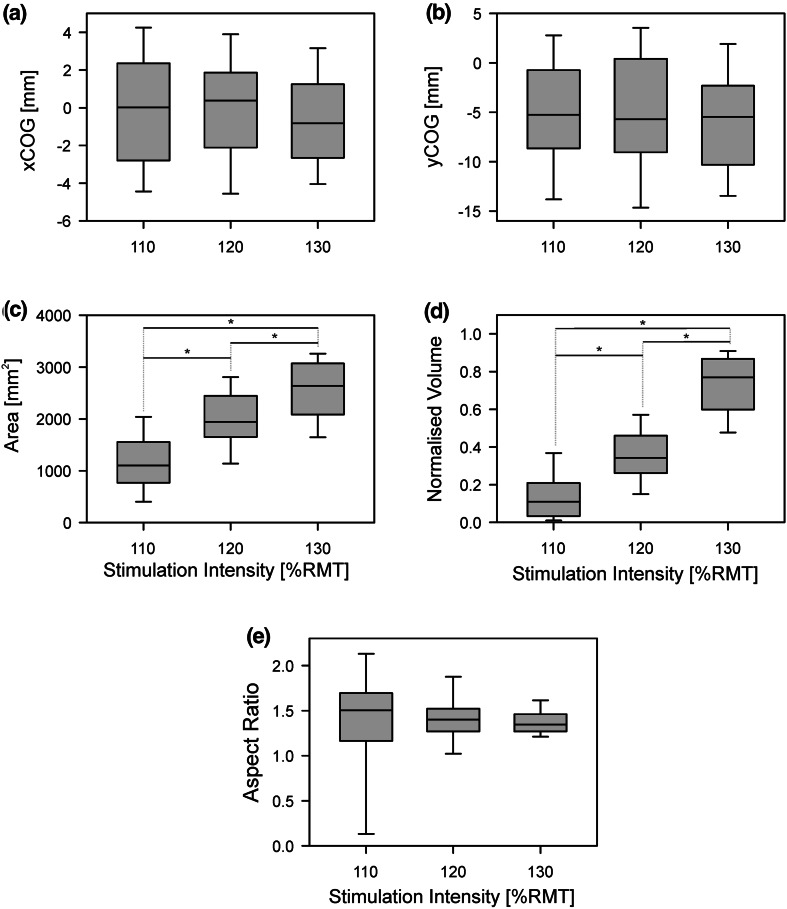
Group data for the effect of stimulation intensity on TMS maps (*n* = 12). Three different stimulation intensities (110, 120 and 130 % of resting motor threshold) were compared. All statistical testing was performed using the non-parametric Friedman Test and any significant difference were further explored using the Wilcoxon Signed-Rank Test. Statistical significance between pairs was declared when *P* < 0.017 (Bonferroni adjusted) and is indicated by *. **a**, **b** Group data for both x- and y-coordinate of the centre of gravity. No effect was found for stimulation intensity. **c**–**e** Group data for the effect of stimulation intensity on map area, map volume and aspect ratio. A significant effect of stimulation intensity on map area and volume was found with all pairs being significantly different. No effect was found for stimulation intensity on the aspect ratio

Across all participants, no difference was observed for either the x- or y-coordinate of the COG [xCOG: χ^2^(2) = 1.17, *P* = 0.56; yCOG: χ^2^(2) = 0.50, *P* = 0.79; Fig. 3a, b]. Map area and volume were both significantly increased with stimulation intensity [area: χ^2^(2) = 22.17, *P* < 0.01; volume: χ^2^(2) = 24.00, *P* < 0.01). For both area and volume, post-hoc testing showed all pairwise comparisons were significantly different using the Bonferroni adjusted *P*-value (0.017) (Fig. 3c, d). Finally, the aspect ratio was analysed. No significant effect of stimulation intensity on the aspect ratio was found [χ^2^(2) = 0.17, *P* = 0.92; Fig. 3e]. Therefore, it can be concluded that the map area increased with stimulation intensity without affecting its shape.

### Experiment 2: Effect of Muscle Activation

**Fig. 4:**
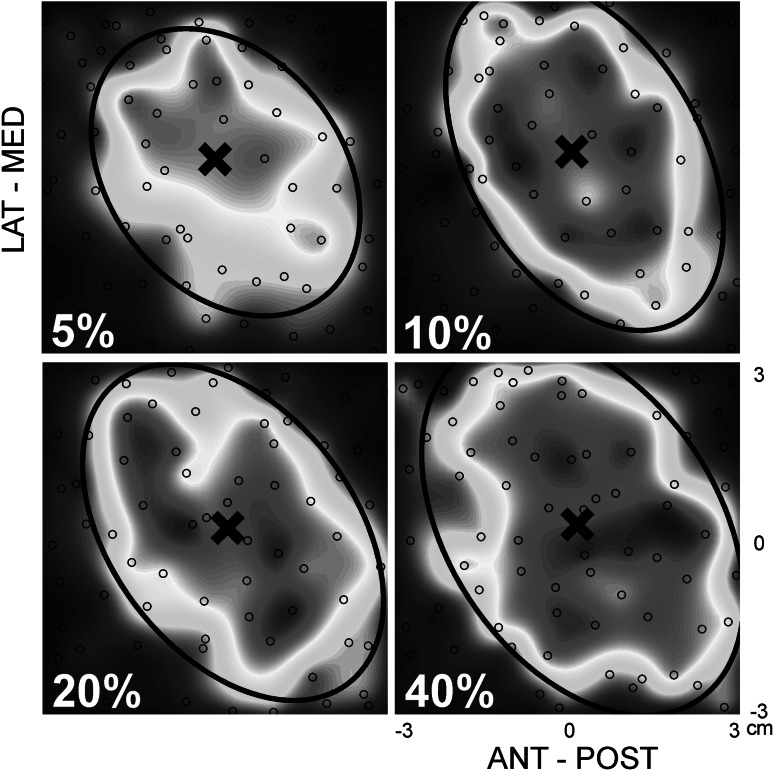
Single participant data illustrating TMS maps acquired at all levels of muscle activation [5, 10, 20 and 40 % of maximum voluntary contraction (MVC)] using a 6 × 6 cm grid, 80 stimuli with an interstimulus interval of 1.5 s at 120 % of resting motor threshold. Each *black open circle* represents one stimulus. The size of the approximated MEP_pp_ is indicated by the * colour*, with * blue* representing a small MEP_pp_ and * red* representing the greatest MEP_pp_. The *black cross* (×) highlights the centre of gravity. In this participant muscle activation was found not to affect the x- and y-coordinate of the centre of gravity, however map area and volume significantly increased with muscle activation. An ellipse was fitted through the data points representing 30 % of the maximum MEP within the 5 % MVC maps and used to study changes in the shape of the excitable area of the map. No change in the shape of the ellipse was observed (Color figure online)

The effect of muscle activation was studied for four different levels of activity (5, 10, 20 and 40 % of MVC). One data set had to be discarded as the 5 % MVC data was missing, and therefore, the analysis was performed on 11 participants. Maps for all levels of muscle activation from a representative participant are shown in Fig. 4.

In this case an increase in the map area can be observed from both 5–10 % as well as 10–40 % of MVC. There is no clear difference in COG between the different levels of muscle activation. Although the excitable area is increased, its shape seems to be unaffected by muscle activation.

**Fig. 5:**
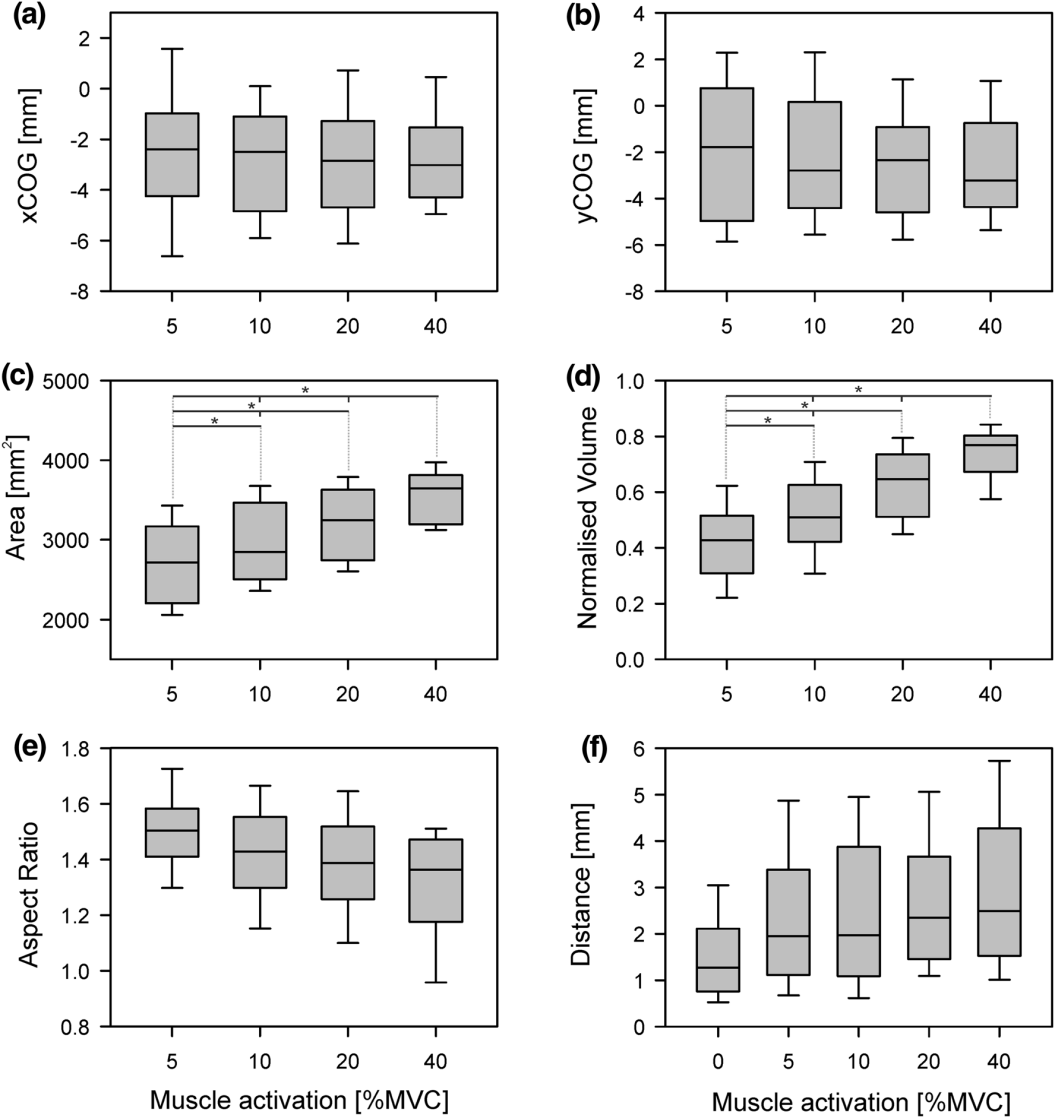
Group data for the effect of muscle activation on TMS maps (*n* = 11). Four levels of muscle activation, 5, 10, 20 and 40 % of maximum voluntary contraction (MVC), were compared. All statistical testing was performed using the non-parametric Friedman Test and any significant difference were further explored using the Wilcoxon Signed-Rank Test. Statistical significance between pairs was declared when *P* < 0.0083 (Bonferroni adjusted) and is indicated by *. **a**, **b** Group data for both x- and y-coordinate of the centre of gravity. No effect was found for muscle activation. **c**, **d** Group data for the effect muscle activation on map area and map volume. A significant effect of muscle activation was found on both map area and map volume. All pairs were found to be significantly different for both the map area and map volume. **e** The maps aspect ratios for different levels of muscle activation. No effect for muscle activation on aspect ratio was found. **f** Group data of the Euclidian distance of each level of muscle activity versus the resting condition

No significant effect was shown for level of muscle activation on the COG (xCOG: χ^2^(3) = 3.55, *P* = 0.31; yCOG: χ^2^(3) = 1.58, *P* = 0.66; Fig. 5a, b). Both map area and volume significantly increased with level of muscle activation [area: χ^2^(3) = 31.91, *P* < 0.01; volume χ^2^(3) = 25.47, *P* < 0.01]. Post-hoc testing showed a significant difference between all pairs for area and volume, for the Bonferroni adjusted *P*-value (0.0083; Fig. 5c, d). Finally, aspect ratio was found to be unaffected by condition [χ^2^(4) = 6.38, *P* = 0.09; Fig. 5e].

The Euclidian distance characterising the distance between all COGs of all active conditions and repetitions compared to the mean COG of the resting condition revealed no effect of condition [χ^2^(4) = 7.49, *P* = 0.11; Fig. 5f].

## Discussion

In this study we demonstrated that map area and volume increase with stimulation intensity and muscle activation, but centre of gravity and shape were unaffected. For both an increased stimulation intensity and higher level of muscle activation, we confirmed the hypothesis that the increased map area reflects a simple scaling of the map.

### The Effect of Stimulation Intensity on the TMS Map

The effect of stimulation intensity on the map’s COG has never been systematically explored. In line with previous studies, area and volume were observed to increase with stimulation intensity (Thordstein et al. 2013). In the present study, both central tendency (COG) and shape (aspect ratio) were invariant to stimulation intensity. It has been suggested that the area of a TMS map is primarily determined by the extent to which the current spreads in the motor cortex (Thickbroom et al. 1998). Therefore, the increase in map area with stimulation intensity might simply be explained by greater activation of the motoneuron pool. With increasing stimulation intensity the increased motoneuron pool activation together with the constant aspect ratio and stable COG, suggests the hand area of the motor cortex is activated symmetrically about the major and minor axes of the stimulation coil.

Stimulating at 130 % of RMT might have induced D-waves by direct activation of the axon hillock (Di Lazzaro et al. 1998a, 2003). In this study it is difficult to unequivocally determine if D-wave recruitment has been present because single stimuli were administered to multiple sites close to, but not specifically over, the motor hotspot and we used a biphasic TMS stimulator which has been reported to result in a less consistent cortical output (Di Lazzaro et al. 2001). Moreover, all recordings at the three different stimulation intensities were performed at rest whilst muscle activation might be needed to evoke a D-wave. This makes it difficult to use the current data to conclude on D-wave recruitment. Nonetheless, it is likely that in some participants we have elicited D-waves during the mapping.

In this study we investigated the cortical representation of the FDI muscle. It is not straightforward that the results presented here do directly translate to the TMS maps of other muscles. Thordstein et al. (2013) reported differences in the effect of stimulation intensity on the map area of the abductor pollicis brevis (APB), the extensor digitorum communis (EDC), the biceps brachii (BB) and the tibialis anterior (TA) muscle, but also highlighted great interindividual differences. Our findings combined with those of Thordstein et al. (2013) highlight that stimulation intensity is an important parameter in TMS mapping and should be carefully considered based on the aim of the mapping procedure and the muscle studied.

### The Effect of Muscle Activity on the TMS Map

In the present study, TMS maps were acquired at four different levels of muscle activation. Whilst it is well documented that MEPs are larger for a muscle that is active compared with a muscle at rest (Hess et al. 1987; Kiers et al. 1993), MEP size does not increase substantially when the muscle is activated above 10 % MVC (Helmers et al. 1989; Taylor et al. 1997). Nonetheless, we found a progressive increase in map area with muscle activation, which contrasted our hypothesis. When comparing a resting and slightly active muscle, the increased excitability is mainly attributed to changes in excitability at the spinal level. This followed from the observation that with muscle activation, stimulation at a level below the cortex did enhance the response amplitudes to a same extent as cortical stimulation (Maertens de Noordhout et al. 1992; Ugawa et al. 1995). These findings have been supported by epidural recordings (Di Lazzaro et al. 1998b; Kaneko et al. 1996a, b). An increase in cortical excitability has also been argued when comparing a resting and slightly active muscle (Mazzocchio et al. 1994). Di Lazzaro et al. (1998b) suggested that an increase in the corticospinal volley might be primarily important when the muscle is contracted at different levels, which is supported by the findings of Ugawa et al. (1995). Nonetheless, based on our results we cannot say if the increased map area is a result of increased spinal or cortical excitability, or a combination of both. However, the contrasting finding of a saturating MEP size and an increased map area does suggest the saturating MEP response might just be a result of the inability of the maximal magnetic stimulus to recruit all cortical neurons to generate greater descending volleys. The progressively increasing map area found in this study shows a greater cortical area is sensitive to eliciting an MEP when the muscle is active. The dissociation between a saturating MEP and increased MEP area might be explained by TMS directly recruiting additional connections (e.g. from the ventral premotor cortex) when the muscle is active. Because this activity will likely be small and temporally dispersed, it might not be readily observable when recording D- and I-waves epidural (Di Lazzaro et al. 1998b).

However, not only greater cortical area with increased excitability can explain the increased map area, as it could also be a result of the stimulation intensity used. Here the approach of Wilson et al. (1995), was adopted maintaining the stimulation intensity at 120 % RMT for all levels of muscle activation. One could argue that because of the 8–10 % reduction in motor threshold and increase in MEP_pp_ amplitude observed for an active muscle versus a muscle at rest (Devanne et al. 1997; Wassermann 2002), it would be straightforward to think map area increases as well. Therefore, the observed increase in map area might be a result of the reduction in motor threshold rather than the cortical excitable area expanding. This viewpoint is supported by the findings of Ngomo et al. (2012), who compensated for the 10 % MSO difference between resting and active muscle motor threshold, and failed to find a difference in the map area between a resting and active muscle. However, in this study we only directly compared the map area at different levels of muscle activation, rather than comparing the map area when the muscle is at rest and active. A minimal change in threshold has been reported when muscle activation exceeds 10 % MVC (Devanne et al. 1997). Therefore, it is unlikely that adjusting the stimulation intensity relative to threshold at every level of muscle activation would have provided different results as those presented here.

When it was first investigated, Wilson et al. (1995) observed a 6 mm mediolateral shift of COG when maps were acquired when the muscle was at rest and activated at 10 % of MVC. However, this was not observed in later studies employing a similar paradigm (Classen et al. 1998; Ngomo et al. 2012). Previously, we reported the COG variability of the adopted mapping method at ±2.4 mm (van de Ruit et al. 2015), which is consistent with other studies where the traditional mapping method was employed (3 mm; Classen et al. 1998; Littmann et al. 2013; Miranda et al. 1997). The statistically insignificant difference of 1 mm in COG between maps acquired with the muscle at rest and all active conditions is an order of magnitude below the inherent variability of the map. Therefore, it can be concluded that in the present study no translation of COG was observed between maps constructed with the muscle at rest or when active.

Lastly, it was observed that the map’s aspect ratio, which was used to define the map’s shape, is indifferent to muscle activation. Combined with the finding of no translation in COG, this suggests a simple scaling of the TMS map area and implies cortical neurons at are equally excitable along the perimeter of the muscle’s cortical representation. Whilst, not statistically significant, Fig. 5e suggests the aspect ratio may decrease with muscle activation. This trend is likely just a consequence of the restricted area that was mapped. The major axis of the ellipse was usually found to be orientated about 45° relative to the anterior-posterior axis, in line with the coil orientation during stimulation. Combined with the notion that the magnetic field is elongated in line with the coil (e.g. Roth et al. 1991; Wilson et al. 1993) the major axis most often covered the full diagonal of the map. Therefore, with increasing muscle activation the major axis could not lengthen, in contrast to the minor axis. As the aspect ratio was calculated by dividing the length of the major axis by the length of the minor axis, this likely explains the decreasing trend.

### Limitations

The mapping method used in the present study uses 80 stimuli delivered pseudorandomly to different locations in a 6 × 6 cm grid with an ISI of 1.5 s (van de Ruit et al. 2015). Using this method, the acquisition time for each map was less than 2 min. As a result, the method allows direct comparison of TMS maps at multiple stimulation intensities and levels of muscle activation whilst keeping the duration of a single session within 2 h. It is unlikely that the use of this method, as compared to a more traditional method using multiple stimuli applied to sites organised on a 1-cm fixed space grid, has affected our results. By adopting the pseudorandom walk method we also minimised any effects of fluctuating corticospinal excitability with time (Ellaway et al. 1998; Kiers et al. 1993) and attention (Rosenkranz and Rothwell 2004; Rossini et al. 1991). As we stimulate with and ISI of 1.5 s, it might be argued that motor cortex excitability might be reduced as is well known to happen with 1 Hz repetitive TMS (Chen et al. 1997). However, the protocols used to reduce excitability deliver at least five times the number of stimuli than are used in the present study. We have recently demonstrated that short trains (180 stimuli) delivered at 1 Hz do not alter motor cortex excitability (Mathias et al. 2014). The likelihood of affecting excitability using an ISI of 1.5 s is further reduced by the fact that stimuli are applied at different sites across the 6 × 6 grid, and the distance between these sites is maximised during the mapping.

The use of a fixed 6 × 6 cm might have affected our results as previous studies have shown map area might exceed 36 cm^2^ (Thordstein et al. 2013; Wilson et al. 1995). The grid size was limited to 6 × 6 cm as we found that when using a larger grid, stimuli would be administered close to and on the temple and ear which caused significant discomfort for the participants. However, in future it would be beneficial to base the grid size on the participant’s head size, to ensure all cortical sites that evoke an MEP are mapped. It is unlikely that the adopted grid size has affected our results as the map area was calculated without the map’s fringe and sites that would evoke an MEP smaller than 10 % of the maximum MEP.

### Implications

As the map area significantly increases with muscle activation and stimulation intensity but the COG and map shape remain the same, this study highlights the importance of choosing experimental conditions and TMS stimulation parameters carefully. This becomes of great importance when using TMS mapping to study brain plasticity in a clinical population (Byrnes et al. 1999; Guerra et al. 2015; Liepert et al. 1999), where fatigue and discomfort are a significant confounding issue. Inadequate parameter selection might lead to the inability to observe a difference in studies investigating changes in corticospinal excitability but also unnecessary participant discomfort. As a result, care should be taken when selecting the parameters for TMS motor mapping and better standardisation of protocols is warranted.

## References

[CR1] Brasil-Neto JP, Cohen LG, Panizza M, Nilsson J, Roth BJ, Hallett M (1992). Optimal focal transcranial magnetic activation of the human motor cortex: effects of coil orientation, shape of the induced current pulse, and stimulus intensity. J Clin Neurophysiol.

[CR2] Byrnes ML, Thickbroom GW, Phillips BA, Wilson SA, Mastaglia FL (1999). Physiological studies of the corticomotor projection to the hand after subcortical stroke. Clin Neurophysiol.

[CR3] Chen R, Classen J, Gerloff C, Celnik P, Wassermann EM, Hallett M, Cohen LG (1997). Depression of motor cortex excitability by low-frequency transcranial magnetic stimulation. Neurology.

[CR4] Classen J (1998). Multimodal output mapping of human central motor representation on different spatial scales. J Physiol-London.

[CR5] Day BL, Dressler D, Denoordhout AM, Marsden CD, Nakashima K, Rothwell JC, Thompson PD (1989). Electric and magnetic stimulation of human motor cortex—surface Emg and single motor unit responses. J Physiol Lond.

[CR6] D’Errico J (2005) Surface fitting using gridfit MATLAB Central File Exchange Retrieved Feb 2012

[CR7] Devanne H, Lavoie BA, Capaday C (1997). Input–output properties and gain changes in the human corticospinal pathway. Exp Brain Res.

[CR8] Di Lazzaro V (1998). Comparison of descending volleys evoked by transcranial magnetic and electric stimulation in conscious humans. Electroencephalogr Clin Neurophysiol.

[CR9] Di Lazzaro V (1998). Effects of voluntary contraction on descending volleys evoked by transcranial stimulation in conscious humans. J Physiol.

[CR10] Di Lazzaro V (2001). Comparison of descending volleys evoked by monophasic and biphasic magnetic stimulation of the motor cortex in conscious humans. Exp Brain Res.

[CR11] Di Lazzaro V, Oliviero A, Pilato F, Mazzone P, Insola A, Ranieri F, Tonali PA (2003). Corticospinal volleys evoked by transcranial stimulation of the brain in conscious humans. Neurol Res.

[CR12] Ellaway PH, Davey NJ, Maskill DW, Rawlinson SR, Lewis HS, Anissimova NP (1998). Variability in the amplitude of skeletal muscle responses to magnetic stimulation of the motor cortex in man. Electromyogr Motor C.

[CR13] Groppa S (2012). A practical guide to diagnostic transcranial magnetic stimulation: report of an IFCN committee. Clin Neurophysiol.

[CR14] Guerra A (2015). Neurophysiological features of motor cortex excitability and plasticity in subcortical ischemic vascular dementia: a TMS mapping study. Clin Neurophysiol.

[CR15] Helmers SL, Chiappa KH, Cros D, Gupta N, Santamaria J (1989). Magnetic stimulation of the human motor cortex: facilitation and its relationship to a visual motor task. Clin Neurophysiol.

[CR16] Hess CW, Mills KR, Murray NM (1987). Responses in small hand muscles from magnetic stimulation of the human brain. J Physiol.

[CR17] Kaneko K, Kawai S, Fuchigami Y, Shiraishi G, Ito T (1996). Effect of stimulus intensity and voluntary contraction on corticospinal potentials following transcranial magnetic stimulation. J Neurol Sci.

[CR18] Kaneko K, Kawai S, Fuchigami Y, Shiraishi G, Ito T (1996). Spinal cord potentials after transcranial magnetic stimulation during muscle contraction. Muscle Nerve.

[CR19] Keel JC, Smith MJ, Wassermann EM (2001). A safety screening questionnaire for transcranial magnetic stimulation. Clin Neurophysiol.

[CR20] Kiers L, Cros D, Chiappa KH, Fang J (1993). Variability of motor potentials-evoked by transcranial magnetic stimulation. Electroencephalogr Clin Neuro.

[CR21] Liepert J, Oreja-Guevara C, Cohen LG, Tegenthoff M, Hallett M, Malin JP (1999). Plasticity of cortical hand muscle representation in patients with hemifacial spasm. Neurosci Lett.

[CR22] Littmann AE, McHenry CL, Shields RK (2013). Variability of motor cortical excitability using a novel mapping procedure. J Neurosci Methods.

[CR23] Maertens de Noordhout A, Pepin JL, Gerard P, Delwaide PJ (1992). Facilitation of responses to motor cortex stimulation: effects of isometric voluntary contraction. Ann Neurol.

[CR24] Mathias JP, Barsi GI, van de Ruit M, Grey MJ (2014). Rapid acquisition of the transcranial magnetic stimulation stimulus response curve. Brain Stimul.

[CR25] Mazzocchio R, Rothwell JC, Day BL, Thompson PD (1994). Effect of tonic voluntary activity on the excitability of human motor cortex. J Physiol.

[CR26] Miranda PC, deCarvalho M, Conceicao I, Luis MLS, DuclaSoares E (1997). A new method for reproducible coil positioning in transcranial magnetic stimulation mapping. Electromyogr Motor C.

[CR27] Ngomo S, Leonard G, Moffet H, Mercier C (2012). Comparison of transcranial magnetic stimulation measures obtained at rest and under active conditions and their reliability. J Neurosci Methods.

[CR28] Pascual-Leone A, Nguyet D, Cohen LG, Brasil-Neto JP, Cammarota A, Hallett M (1995). Modulation of muscle responses evoked by transcranial magnetic stimulation during the acquisition of new fine motor skills. J Neurophysiol.

[CR29] Rosenkranz K, Rothwell JC (2004). The effect of sensory input and attention on the sensorimotor organization of the hand area of the human motor cortex. J Physiol.

[CR30] Rossini PM, Desiato MT, Lavaroni F, Caramia MD (1991). Brain excitability and electroencephalographic activation - noninvasive evaluation in healthy humans via transcranial magnetic stimulation. Brain Res.

[CR31] Rossini PM (1994). Non-invasive electrical and magnetic stimulation of the brain, spinal cord and roots: basic principles and procedures for routine clinical application. Report of an IFCN committee. Electroencephalogr Clin Neurophysiol.

[CR32] Roth BJ, Saypol JM, Hallett M, Cohen LG (1991). A theoretical calculation of the electric-field induced in the cortex during magnetic stimulation. Electroencephalogr Clin Neurophysiol.

[CR33] Rothwell JC, Thompson PD, Day BL, Boyd S, Marsden CD (1991). Stimulation of the human motor cortex through the scalp. Exp Physiol.

[CR34] Taylor JL, Allen GM, Butler JE, Gandevia SC (1997). Effect of contraction strength on responses in biceps brachii and adductor pollicis to transcranial magnetic stimulation. Exp Brain Res.

[CR35] Thickbroom GW, Sammut R, Mastaglia FL (1998). Magnetic stimulation mapping of motor cortex: factors contributing to map area Electromyogr Motor C.

[CR36] Thielscher A, Kammer T (2004). Electric field properties of two commercial figure-8 coils in TMS: calculation of focality and efficiency. Clin Neurophysiol.

[CR37] Thordstein M, Saar K, Pegenius G, Elam M (2013). Individual effects of varying stimulation intensity and response criteria on area of activation for different muscles in humans. A study using navigated transcranial magnetic stimulation. Brain Stimul.

[CR38] Ugawa Y, Terao Y, Hanajima R, Sakai K, Kanazawa I (1995). Facilitatory effect of tonic voluntary contraction on responses to motor cortex stimulation. Electroencephalogr Clin Neurophysiol.

[CR39] Uy J, Ridding MC, Miles TS (2002). Stability of maps of human motor cortex made with transcranial magnetic stimulation. Brain Topogr.

[CR40] van de Ruit M, Perenboom MJ, Grey MJ (2015). TMS brain mapping in less than two minutes. Brain Stimul.

[CR41] Wassermann EM (2002). Variation in the response to transcranial magnetic brain stimulation in the general population. Clin Neurophysiol.

[CR42] Wassermann EM, Mcshane LM, Hallett M, Cohen LG (1992). Noninvasive mapping of muscle representations in human motor cortex. Electroencephalogr Clin Neurophysiol.

[CR43] Wilson SA, Thickbroom GW, Mastaglia FL (1993). Transcranial magnetic stimulation mapping of the motor cortex in normal subjects—the representation of 2 intrinsic hand muscles. J Neurol Sci.

[CR44] Wilson SA, Thickbroom GW, Mastaglia FL (1995). Comparison of the magnetically mapped corticomotor representation of a muscle at rest and during low-level voluntary contraction. Electroencephalogr Clin Neurophysiol.

